# Integrated Cancer Subtyping using Heterogeneous Genome-Scale Molecular Datasets

**Published:** 2020

**Authors:** Suzan Arslanturk, Sorin Draghici, Tin Nguyen

**Affiliations:** Department of Computer Science, Wayne State University Detroit, MI 48202, USA; Department of Computer Science, Wayne State University Detroit, MI 48202, USA; Department of Computer Science and Engineering, University of Nevada Reno, Nevada 89557, USA

**Keywords:** data integration, disease subtype discovery, omics data

## Abstract

Vast repositories of heterogeneous data from existing sources present unique opportunities. Taken individually, each of the datasets offers solutions to important domain and source-specific questions. Collectively, they represent complementary views of related data entities with an aggregate information value often well exceeding the sum of its parts. Integration of heterogeneous data is therefore paramount to i) obtain a more unified picture and comprehensive view of the relations, ii) achieve more robust results, iii) improve the accuracy and integrity, and iv) illuminate the complex interactions among data features. In this paper, we have proposed a data integration methodology to identify subtypes of cancer using multiple data types (mRNA, methylation, microRNA and somatic variants) and different data scales that come from different platforms (microarray, sequencing, etc.). The Cancer Genome Atlas (TCGA) dataset is used to build the data integration and cancer subtyping framework. The proposed data integration and disease subtyping approach accurately identifies novel subgroups of patients with significantly different survival profiles. With current availability of vast genomics, and variant data for cancer, the proposed data integration system will better differentiate cancer and patient subtypes for risk and outcome prediction and targeted treatment planning without additional cost and precious lost time.

## 1. Introduction

Genomic and epidemiologic studies over the past decade have generated a wealth of data, including molecular, variant, and clinical data on both individuals and populations that can be leveraged to better understand cancer risk, progression, and outcomes. Subtyping patient disease populations using high-dimensional molecular data has transformed how researchers and clinicians interpret and quantify heterogeneity within a disease. Subtyping has been highly effective in discovering cancer types, tumor histologies, survival rates, treatment planning and responses. Investigation of clinically relevant disease subtypes cannot be achieved by using a single dataset in isolation from others due to the heterogeneity of cancer with multifactorial etiology. Hence, careful integration of diverse data is crucial (e.g. molecular, clinical, environmental data) [1].

The heterogeneity of diseases such as breast cancer is well recognized and gene expression profiling has been used to identify at least four major subtypes: luminal A, luminal B, HER2+ and basal-like [2]. In the past decade, important clinical advances in cancer treatments are attributed to molecularly targeted treatments aiming at specific genes such as estrogen receptor alpha (ER-α), the human epidermal growth factor receptor 2 (HER2), the epidermal growth factor receptor (EGFR), etc [3]. Targeted treatments result in greater efficacy and fewer debilitating or dose limiting side effects. This clearly proves that it is important to identify and appropriately treat each individual disease subtype. However, our current understanding of disease subtypes appears to be very limited. Despite targeted treatment advances, targeted therapies often fail for some patients. For breast cancer, while 20% of tumors overexpress the HER2 oncogene, one-third of these fail to show response to HER2-targeted therapies right from the outset. Research and clinical studies present a similar story for anti-estrogen treatment of ER-α-positive breast cancer, and androgen ablation of androgen receptor positive prostate cancer [4]. Not all patients show an initial response, and from those who do, a significant number will develop resistance. The fact that a substantial fraction of patients with a given subtype of cancer respond very differently to the same treatment, either immediately or later on, means that either: **i) the known subtypes are not truly homogeneous and must be further refined, or ii) that subgroups of patients may have different mechanisms of defense against the same tumor type.**

Several studies have been undertaken to determine disease subtypes. Agglomerative hierarchical clustering (HC) [5, 6], model-based approaches [7, 8], graph theoretical methods [9, 10], matrix factorization [11] and neural networks [12, 13] are widely used techniques to identify the heterogeneity within a disease. Subtypes of cancer can be identified using different data types such as clinical data, DNA sequencing, miRNA sequencing, protein expression, mRNA sequencing, DNA Methylation, somatic variants [14, 15, 20, 24, 31].

Consensus Clustering (CC) [16] is a state-of-the-art approach desired to find a single clustering by reconciling clustering information from various sources or from different runs of the same algorithm. However, CC cannot be used to combine multiple data types with different scales and most of the time the analysis of each data type leads to different results (subgroups) that are hard to interpret. Other machine learning approaches such as iCluster [17], and iClusterPlus [18] addresses the challenge of integration by using statistical models that can simultaneously perform clustering, data integration, feature selection and dimensionality reduction using a probabilistic matrix factorization approach. Though powerful, they are limited by their strong assumptions about the data as well as by the gene selection step necessary to reduce computational complexity. Similarity Network Fusion (SNF) [19] is another state-of-the-art approach that can be used for cancer subtyping by integrating multiple data types. Herein, samples are constructed into separate networks for each data type and fused into one network that represents the full spectrum of the data. However, the unstable nature of kernel-based clustering makes the algorithm sensitive to small changes in molecular measurements or in its parameter settings. Cancer Integration via Multikernel Learning (*CIMLR*) is another kernel-based approach that adds weights to different data types [30].MaxSilhoutte is a clustering technique based on cluster tightness and separation where each cluster is represented by a so-called silhouette [27]. MaxSilhoutte, however, is not designed to integrate multiple data types, and hence requires the separate datasets to be concatenated for integrative analysis.

Nguyen et. al.’s recent paper [20] has inspired and given us the basis on which we have built a data integration and disease-subtyping framework. They have proposed a novel integrative approach, called Perturbation clustering for data INtegration and disease Subtyping (PINS) that addresses subtype discovery using a single datatype or integration of multiple data types. The method determines the optimal number of clusters and then partitions the samples in a way such that the results are robust to noise and data perturbation. The study integrated multiple quantitative numerical data types (mRNA, methylation, microRNA) that came from different platforms, different scales and different cellular phenomena. Though powerful, the approach proposed here can only be applied on quantitative numerical data types. In this paper, we have proposed a new method that can integrate both qualitative and quantitative numerical data to better identify the cancer subtypes and novel subgroups of patients with significantly different survival profiles.

Cancer being a heterogeneous disease with large genetic diversity even between tumors of the same cancer types, it is common for the patients to have significant differences between their molecular profiles. Hence, majority of the recent studies use integrative approaches that combines multiple types of molecular data such as Methylation, mRNA expression, DNA copy number variation etc. accounting for variations among individuals and thereby achieving more accurate subtyping [21, 28, 29]. However, because of the noise level of these datasets and the complexity of the disease, the results are not producing significant separation between the subgroups [22]. Therefore, recent studies have proposed to use additional datasets such as somatic variants [21, 23], and clinical data [30] in combination with the aforementioned molecular data types as a new source of information. Gligorijevic et. al. has shown that careful integration of different data types can address several challenges as i) stratification of patients with different clinical outcomes, ii) prediction of driver genes, iii) repurposing of drugs treating particular cancer patient groups [21].

In a previous study, we have proposed a cancer subtyping methodology using solely somatic variant data available at TCGA [24]. We were not interested in any clusters that form or disappear due to small changes in the data, but rather for those groupings that remain stable across many clusterings built in the presence of small changes. To identify such clusters, we have generated new datasets by perturbing the original data using a Post Randomization (PRAM) method and reconstructing the clustering. The discrepancy between the original and the perturbed data was used to assess the stability of the clusters. The results have shown that the proposed approach can identify disease subtypes better than the state-of-the-art approaches.

In this paper, we integrated the subtyping approach we proposed in [24] for somatic variants with the subtyping approach defined by two of our authors [20] for mRNA, miRNA and Methylation. We developed a data integration system for cancer subtyping that flexibly integrates both qualitative and quantitative datatypes using existing datasets available at TCGA. We believe that integrating multi-variate heterogeneous datatypes will improve the consistency and actionable information value of the consensus subtypes. Developed framework will be a valuable precision medicine resource for the wider scientific community on other diseases to pursue a multitude of studies that have not been possible due to limitations of existing integrative subtyping methods.

## 2. Methods

We analyzed five different cancers available at The Cancer Genome Atlas (TCGA) website (https://tcga-data.nci.nih.gov/): Kidney Renal Clear Cell Carcinoma (KIRC), glioblastoma multiforme (GBM), acute myeloid leukemia (LAML), breast invasive carcinoma (BRCA), and colon adenocarcinoma (COAD). Table 1 shows the basic descriptions of the five datasets we have analyzed. We used mRNA expression, DNA Methylation, miRNA expression and somatic variant data to identify the subtypes for each of the five cancers. Subtyping is first performed on each data in isolation and the obtained results are then integrated to improve the differentiation between subgroups.

### 2.1. Subtyping Qualitative Data

Herein, we have used the somatic variant data to identify the cancer subtypes. The somatic variant data is stored in a binary matrix, where “1” denotes a mutation on the host gene, and “0” denotes the absence of mutation, with the rows and columns corresponding to the samples and genes, respectively. Somatic variants can be defined as an alteration in DNA identified by comparing a normal sample with a tumor sample and generally very sparse since the proportion of variants are minor compared to the whole genome size.

For each of the five datasets, we calculated the pairwise distance between all patients using the Jaccard index. For each patient, the somatic variant profile is represented as a binary vector and the Jaccard index is computed as

(1) $$J(A,B)=M_{11}/\left(M_{11}+M_{01}+M_{10}\right)$$

*M*_11_ represents the total number of mutated genes for patients *A* and *B*, *M*_01_ represents the total number of genes where patient *A* has a value of ‘0’ and *B* has a value of ‘1’, and *M*_10_ represents the total number of genes where patient *A* has a value of ‘1’ and *B* has a value of ‘0’. The Jaccard indexis computed for each pair of patient in the dataset, resulting in a similarity matrix that can be used as an input to any distance based clustering method. To identify the subtypes, we exploit the agglomerative hierarchical clustering using the Ward’s method as the linkage criteria as well as the Partition Around Medoids (PAM) clustering. The identified subtypes can be illustrated in a matrix form referred to as the connectivity matrix.

#### 2.1.1 Construction of Data Connectivity

The input is a dataset, $E\in R^{NXM}$ where *N* is the number of subjects and *M* is the number of features for each subject. For somatic variants, the pairwise similarity between each pair of subject is computed using the Jaccard similarity measure and stored in a matrix form. We then partition the subjects into *k* clusters for each value of k∈[2..K] using a clustering algorithm. We have used the Agglomerative Hierarchical Clustering and PAM but a number of other classical distance based clustering approaches could be used instead. The input dataset *E* can be presented as a set of vectors $E=\left\{e_{1},e_{2},\ldots ,e_{N}\right\}$, where each vector *e*_*i*_ represents the features of the *i*^*th*^ subject. A partition $P_{k}=\left\{P_{1},P_{2},\ldots ,P_{k}\right\}$ represents a set of subjects that are members of the same cluster. We generate a pairwise connectivity matrix $C_{k}\in {\left\{0,1\right\}}_{NXN}$, which can be defined as follows:

(2) $$C_{k}(i,j)=\{\begin{array}{cc} 1 & \text{if}\;\exists t\in [2..K]:e_{i},e_{j}\in P_{t}\\ 0 & \;otherwise\; \end{array}$$

Here, the connectivity between two subjects is ‘1’ if and only if they belong to the same cluster and ‘0’ otherwise.

#### 2.1.2. Generating Perturbed Datasets

One challenge of clustering is the determination of the number of clusters, i.e. the number of subtypes. The proposed approach hypothesizes that the number clusters should be robust with respect to the systemic noise of the features within the population. Hence, we have utilized a perturbation mechanism to add noise to the input data many times and construct connectivity matrices for each perturbed dataset. The original and the average perturbed connectivity matrices are then compared to assess the stability of pair-wise connectivity (identical or different cluster membership) for each pair of subjects. Number of clusters, providing the highest degree of stability with a certain amount of perturbation, is considered to be optimal.

Accordingly, we first developed a perturbation method for discrete and binomial data by employing a post-randomization (PRAM) methodology [25]. PRAM is a perturbative method for disclosure protection of qualitative variables [25]. Applying PRAM on a dataset leads the values of a number of variables to be changed according to a specified probability mechanism. PRAM is commonly used to protect sensitive data files against disclosure by randomization of individual record data with the proper choice of transition probabilities.

As a first step in perturbing data, let *ε* denote a qualitative variable in the original dataset with *K* categories, numbered 1,..,K to which PRAM is applied and $\hat{\varepsilon }$ denote the same categorical variable in the perturbed data file. Let $P=\left\{p_{kl}\right\}$ be a KxK Markov probability matrix defined as; $P(\hat{\varepsilon }=l|\varepsilon =k)$, that denotes the probability of the original value *ε* = *k* transitioned into a value of $\hat{\varepsilon }=l$. For a dataset of *n* records, let *ε*^(*r*)^denote the value of *ε* for the *r*th record in the data file.

Given that $\varepsilon^{\left(r\right)}=k$, applying PRAM means that the value of ${\hat{\varepsilon }}^{\left(r\right)}$ is drawn from the probability distribution $p_{k1},\ldots ,p_{kK}$ This procedure can then be applied independently on each record in the datafile. Consider an example where the variable *ε* represents the somatic variants with “1” denoting a mutation on the host gene, and “0” denoting the absence mutation (hence, the number of categories, *K* = 2). The Markov probability matrix *P* can then be defined as follows:

(3) $$P=\left(\begin{array}{rr} \theta_{0}\; & 1-\theta_{0}\\ 1-\theta_{1} & \theta_{1} \end{array}\right)$$

In this paper, we have randomized the variants for each subject using equal probabilities for the transitions. A variant will switch from present to absent and vice versa, with the probability of *θ*, and stay as is with a probability 1 – *θ*. If the transition probabilities are set too low, the added noise will not perturb the data sufficiently. If the probabilities are too high, the perturbation may significantly change the patterns of the data, causing the subtypes to be indifferentiable due to the added noise. Therefore, the selection of the transition probabilities has an important effect on identifying the hidden subtypes of the data. To determine the transition probabilities, we considered the mutation in each gene as an independent Bernoulli trial. The Bernoulli process applies to discrete stochastic sequences and each component (1,0) designates whether a mutation happened at a specific position. This way, we can use the variance of each Bernoulli trial to determine the transition probabilities of PRAM.

(4) $$\sigma^{2}=median\left\{\sigma_{1}^{2},\ldots ,\sigma_{M}^{2}\right\},\text{where}\;\sigma_{j}^{2}=var\{E(i,j),i\in [1..N],j\in [1..M]\}.$$

In equation 4, the variance, *σ*^2^, would correspond to 1 – *θ*, e.g. if the median variance of a dataset is calculated as 0.02, then the transition probability, *θ*, is set to 0.98, which means that there would be a 98% probability for any somatic variant (0,1) at ***E***(*i, j*)to remain the same. This process allows us to construct numerous perturbed versions of the original data.

#### 2.1.3. Construction of Perturbed Connectivity

To construct the connectivity matrices for each perturbed data, we clustered each perturbed dataset using both hierarchical clustering and PAM with varying values of k∈[2..1]. Since true cluster assignments is assumed to be robust with respect to small perturbations, the ideal case would be the individual patient’s cluster assignments to remain the same on both original and perturbed datasets for the optimal cluster size, *k.* Since we have generated many perturbed versions of the original data (say *L* perturbation datasets) for each cluster *k,* the overall connectivity matrix, *A*_*k*_ can be calculated by averaging the connectivity matrices of each perturbed dataset, $G_{k}^{1},G_{k}^{2},\ldots ,G_{k}^{l}$ where l∈[1..L]. $G_{k}^{1}$ *and A*_*k*_ can be defined as follows:

(5) $$G_{k}^{l}(i,j)=\{\begin{array}{rr} 1 & \;if\;i\;and\;j\;belong\;to\;the\;same\;cluster\;\\ 0 & \;otherwise\; \end{array}$$

(6) $$A_{k}=\frac{1}{l}\sum_{l=1}^{l}G_{k}^{l}$$

Hence, the discrepancy between the original connectivity matrix $C_{k}\in {\left\{0,1\right\}}_{NXN}$ and the average connectivity matrix of the perturbed data *A*_*k*_ can be calculated to measure the stability of each cluster size *k.* The cluster associated with the minimal discrepancy is then identified as the optimal cluster size.

### 2.2. Subtyping Quantitative Data

Herein, we have used mRNA expression, DNA methylation and microRNA data to identify the cancer subtypes. We have used the method introduced by Nguyen et. al. [20]. Each of these are quantitative numerical datatypes with different scales. Each datatype is first used in isolation to identify the subtypes and the results are then integrated to determine the consensus subtypes. The perturbation methodology used for quantitative data is different from the method used for qualitative data.

#### 2.2.1. Construction of Data Connectivity

The input is a dataset,$E\in R^{NXM}$ where *N* is the number of subjects and *M* is the number of features for each subject. We partition the subjects into *k* clusters for each value of k∈[2..K] using the traditional k-means clustering. The connectivity matrices for the quantitative data are then constructed the same way as in qualitative data (See Section 2.1).

#### 2.2.2. Generating Perturbed Datasets

For the quantitative data, the perturbation is performed by adding Gaussian noise to the original data. We perturb the data with a noise level that has a variance equal to the variance of the data in order to prevent the perturbation from significantly changing the patterns of the data and causing the subtypes to be indifferentiable due to the added noise. The variance is calculated as follows:

(7) $$\sigma^{2}=median\left\{\sigma_{1}^{2},\ldots ,\sigma_{M}^{2}\right\},\text{where}\;\sigma_{i}^{2}=var\{E(i,j),\;i\in [1..N],\;j\in [1..M]\}.$$

We then generate *H* new datasets (e.g. 200), $L^{h}\in R^{NxM},h\in [1..H]$ by adding Gaussian noise $N\left(0,\sigma^{2}\right)$ to the original data.

(8) $$L^{h}=E+N\left(0,\sigma^{2}\right)$$

Each perturbed data *L*^*h*^ is then re-clustered for each cluster size. The perturbed connectivity matrices for quantitative and qualitative data are constructed using the same approach as discussed in Section 2.1.2.

### 2.3. Integration of Connectivity Matrices

Once the connectivity matrices for the optimal cluster size *k* are generated for each datatype, we then integrate those matrices by the method described below. In the ideal case, different data types should give consistent connectivity between subjects. However, in practice, different data types can give contradictory information. Therefore, we need to rely on the average connectivity between data types in order to partition the samples. The average pairwise connectivity between samples can be calculated as follows:$S_{c}=\left(\sum_{i=1}^{T}C_{i}\right)/T$, where *T* represents the different datatypes within the dataset. Hence, *S*_*c*_(*i*, *j*) will be 0 if *i* and *j* are never clustered together , 1 if *i* and *j* are always clustered together, and between 0 and 1, if *i* and *j* are clustered together in some datatypes.

**Figure 1.**
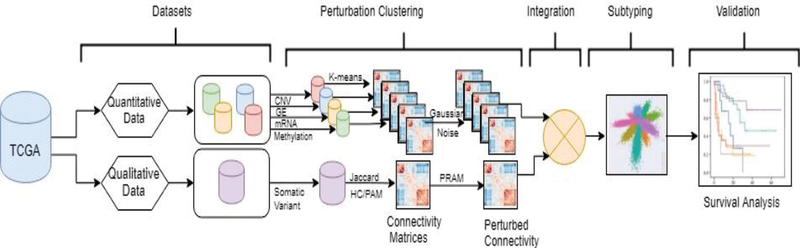
Framework of the proposed subtyping and data integration method

We refer to *S*_*c*_ as the similarity matrix and (1 — *S*_*c*_) as the distance matrix. The matrix of pairwise distances (1 – *S*_*c*_) can be directly used by a similarity-based clustering algorithm such as agglomerative hierarchical clustering, PAM or dynamic tree cut to partition the dataset. The framework of the proposed data integration and disease subtyping methodology is illustrated in Figure 1.

#### 2.3.1. Further Splitting Discovered Groups

At this stage, we attempt to sub-split the discovered subgroups to better identify the clusters. Given that the subgroup identification proposed here is an unsupervised approach, prior information such as patient demographics that may be predominant are missing. The presence of a subgroup can therefore be obscured. In addition, there may be distinct subgroups that share clinically relevant characteristics. For instance the already identified subgroups of breast cancer, Luminal A and Luminal B, are both estrogen receptor positive, which may require the two groups to be further examined to identify the heterogeneity between them. First, we check the agreement between the constructed connectivity matrices of each data type. An entry will be ‘0’ if the pair of subjects, *i and j* are never clustered together and ‘1’ if they are always clustered together. If the pair is clustered together only within the connectivity matrices of certain datatypes, we consider no agreement between the two subjects. If there is an agreement that exceed the set threshold (e.g., >50%), we consider further splitting the subgroups into clusters.

(9) $$agreement\;=\left(Card\left\{S_{c}(i,j)=0\vee S_{c}(i,j)=1,i<j\right\}\right)/\left(\begin{array}{l} N\\ 2 \end{array}\right)$$

In order to sub-split the identified subgroups we have used the gap statistics. Gap statistic is a method used to estimate the most possible number of clusters in a partition clustering. We have used the criterion introduced by Tibshirani et. al. [26] that uses the output of any clustering algorithm by comparing the change in within-cluster dispersion with that expected under an appropriate reference null distribution. Suppose *D*_*r*_ be the sum of the pairwise distances for all points in cluster *r* and *n* be the sample size, then;

(10) $$W_{k}=\sum_{r=1}^{k}\frac{1}{2n_{r}}D_{r}.$$

*W*_*k*_ can be defined as the within-cluster sum of squares around the cluster means. The gap statistic can then be computed as

(11) $$Gap_{n}(k)=E_{n}^{*}\left\{\log\left(W_{k}\right)\right\}-\log\left(W_{k}\right).$$

where $E_{n}^{*}$ denotes the expectation under a sample of size *n* from the reference distribution. We have applied the gap statistic only on subgroups that have at least a certain number of subjects (e.g., 30). The subgroup(s) are split into *k* clusters with varying values of k∈[1…K/2]. Note that, if the optimal number of clusters using the Tibshirani criterion is 1, no further splitting would be required. If otherwise, the subgroup would be further split into *k* clusters. One limitation of further splitting the subgroups is the potential of overfitting. As the within-cluster similarity increases when forming new and finer clusters, it may also lead to fitting the noise. In order to prevent the overfitting, we introduced a regularization term that restricts high number of clusters by reducing the gap ratio each time a new cluster is introduced.

## 3. Results

The results of the proposed method using five different datasets is reported in Table 2, where ***k*** denotes the optimal cluster size and ***Cox P*** denotes the statistical significance between identified subtypes estimated based on the predictive accuracy on the survival time. The subtypes are analyzed using the Kaplan-Meier analysis and their statistical significance is assessed using Cox regression. The integrated results clearly show a better differentiation than the individual data types.

**Figure 2.**
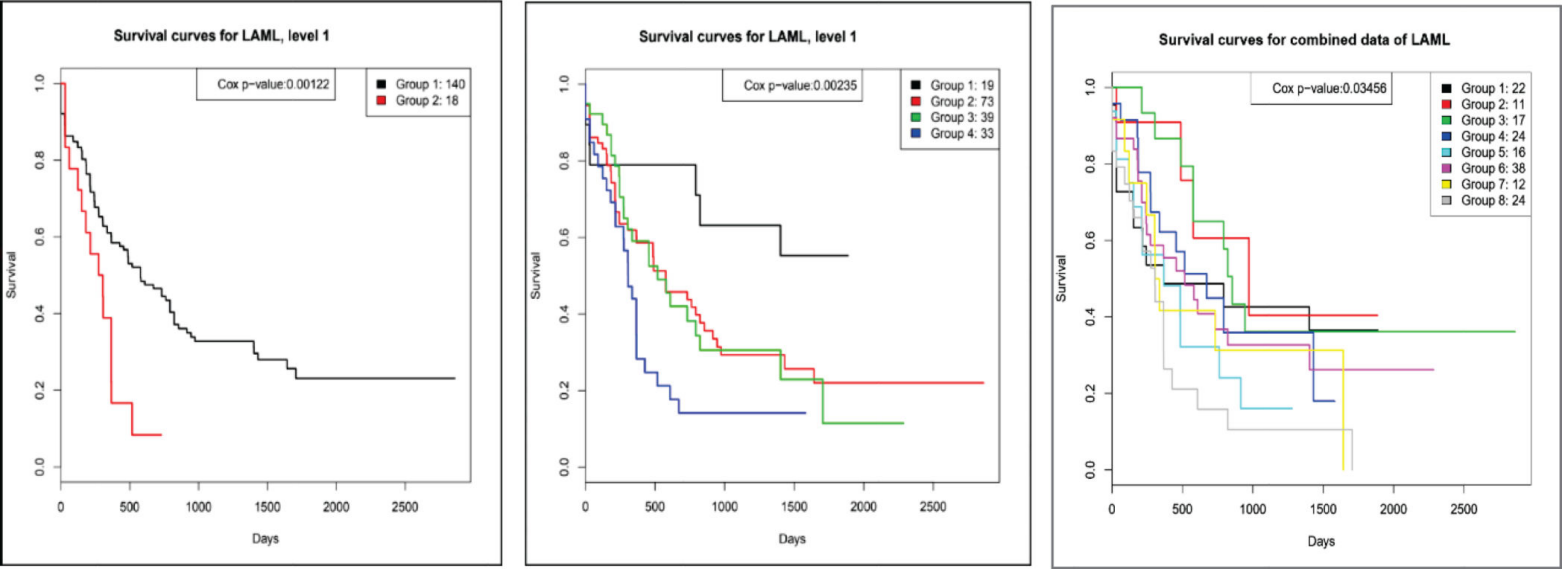
Kaplan-Meier survival curves of integrative genomic data clustering using proposed approach (left), PINS (center) and CC (right).

Our results are compared with PINS, CC, SNF, iCluster+ and maxSilhoutte methods. The results have shown that the proposed integration significantly differentiates the identified subtypes for all investigated diseases and outperforms the integrated results of the aforementioned state-of-the-art techniques. Figure 2 (left) shows the Kaplan-Meier survival curves of the proposed methodology using the acute myeloid leukemia (LAML) dataset compared with the survival curves obtained through integration of mRNA, methylation and miRNA data using PINS (center) and CC (right). The proposed integrative clustering with somatic variants, methylation, mRNA and miRNA discovers two patient groups with significantly different survival profiles (p-value = 10^−3^). In contrast, the integrative clustering without somatic variants discovers four different patient groups with less significant survival profiles (p-value = 2.4×10^−3^). These survival curves clearly show that incorporating qualitative data (i.e. somatic variants) into the integration process outperforms the subtyping performance. We observed similar performances on the other datasets.

### 3.1. Further Analysis of Discovered Subtypes

Herein, we looked into significant survival differences to identify cancer subtype specific biomarkers. Specifically, we investigated mutations that are abundant in patients within the short-term survival group but not within the long-term survival group and vice versa. For LAML, mutated genes that are abundant in patients within the short-term survival group are *TP53, DNMT3A* and *FLT3* while *NPM1* is found to be enriched in long-term survival groups. *VHL* is mutated in all subgroups of KIRC except one in which all patients survive at the end of the study. Similarly, *PBRM1* is found to have high mutation rates on patients with short-term survivals and low mutation rates on patients with long-term survivals. Our results show that GBM subtypes are highly influenced by methylation profiles (See Table 2). The genes identified as biomarkers in GBM are *TTN, TP53, PTEN* and *EGFR.* The mutation rates of those genes are significantly higher in patients that have short-term survival rates. *IDH1* and *ATRX* are highly enriched in patients with long-term survival. Parsons et. al. have indeed shown that patients with *IDH1* mutation usually have a significantly longer survival [27] and IDH1 can be used as target for therapy and drug development [28]. High mutations in *BRAF* and *p53* were determined in patients with short-term survival of COAD. Contrary to recent studies, no significant association was found between *KRAS* and short-term survival [29]. We have further compared the BRCA subtypes with known targets. Out of 172 patients, there are 34 ER-negative (ER-), 134 ER-positive (ER+) and 4 not evaluated. 27 out of 34 ER- patients are found to have a short-term survival, whereas the ER+ patients are uniformly distributed across the four clusters identified.

## 4. Summary and Conclusion

In this paper, we have identified cancer subtypes using somatic variant, mRNA, methylation, and miRNA data types. This method can be applied on any quantitative or qualitative dataset for the purpose of disease categorization, patient subgroup detection, response to treatment identification, drug development and repurposing, or biomarker detection. This method can be applied on any dataset for the purpose of disease categorization, patient subgroup detection, response to treatment identification, drug development and repurposing, or biomarker detection. The method scales well to high dimensional data. However, the time complexity is higher as compared to classical approaches due to repeated perturbations. This can be resolved by performing the computations in parallel. Another limitation of the proposed method is that all data types are treated equally in determining subtypes, which may not always be appropriate. For instance, studies have shown that methylation plays a major role in determining the GBM subtypes. One way to address this limitation is to combine the connectivity matrices in a weighted manner. Future work includes: i) incorporating different mutation types (silent, missense, nonsense, etc.), classifications (SNP, insertion, deletion, etc.) and counts into the proposed disease subtyping method ii) incorporating clinical data into the integration process to examine the significance of different survival profiles and iii) utilizing the identified biomarkers to measure pathway deregulations, which would justify the application of certain therapies and customize treatment plans for individuals.

## Figures and Tables

**Table 1.** Description of the five datasets from The Cancer Genome Atlas (TCGA)

Data Set	Patients	Data Type	Components no.

**KIRC**	124	mRNA	17,974
		Methylation	23,265
		miRNA	590
		Somatic Variant	3412

**GBM**	273	mRNA	12,042
		Methylation	22,833
		miRNA	534
		Somatic Variant	5172

**LAML**	158	mRNA	16,818
		Methylation	22,833
		miRNA	552
		Somatic Variant	1259

**BRCA**	172	mRNA	20,100
		Methylation	22,533
		miRNA	718
		Somatic Variant	8805

**COAD**	145	mRNA	17,062
		Methylation	24,454
		miRNA	710
		Somatic Variant	13,309

**Table 2.** Comparison of the subtypes identified using the proposed method and state-of-the-art techniques. Cells highlighted in green have Cox P-values < 0.01. Cells highlighted in yellow have Cox P-values between 0.01 and 0.05.

		*Proposed*		*PINS*		*CC*		*SNF*		*iCluster*+		*maxSilhoutte*	

Name	Data type	k	Cox P	k	Cox P	k	Cox P	k	Cox P	k	Cox P	k	Cox P

**GBM**	mRNA	2	0.408	2	0.408	5	0.281	2	0.992	10	0.056	2	0.408
	Methylation	2	10^−4^	2	10^−4^	6	0.001	2	0.017	10	0.003	3	10^−4^
	miRNA	3	0.051	4	0.086	6	0.526	2	0.401	10	0.09	2	0.276
	Somatic Variant	2	0.016	-	-	-	-	3	0.632	8	0.324	-	-
	**Integration**	**6**	**4×10**^**−5**^	**3**	**8.7×10**^**−5**^	7	0.039	4	0.162	5	0.156	2	0.408

**LAML**	mRNA	6	0.003	5	0.003	6	8×10^−4^	2	0.327	6	0.01	2	0.027
	Methylation	4	0.893	6	0.239	7	0.049	2	0.993	10	0.002	2	0.04
	miRNA	2	0.065	2	0.072	6	0.017	3	0.183	-	-	2	0.07
	Somatic Variant	6	0.469	-	-	-	-	3	0.532	5	0.324	-	-
	**Integration**	**2**	**10**^**−3**^	**4**	**2.4×10**^**−3**^	8	0.035	3	0.027	5	0.036	3	0.032

**BRCA**	mRNA	2	0.902	2	0.902	8	0.114	2	0.969	9	0.101	2	0.902
	Methylation	4	0.048	4	0.048	8	0.578	5	0.878	10	0.083	2	0.702
	miRNA	3	0.218	3	0.218	5	0.142	2	0.105	-	-	2	0.093
	Somatic Variant	2	0.002	-	-	-	-	3	0.324	10	0.132	-	-
	**Integration**	**4**	**3×10**^**−4**^	7	**3.4×10**^**−2**^	7	0.667	2	0.398	10	0.402	2	0.902

**COAD**	mRNA	2	0.109	2	0.113	8	0.048	2	0.148	6	0.29	2	0.113
	Methylation	2	0.719	2	0.741	8	0.034	2	0.389	10	0.194	2	0.741
	miRNA	4	0.468	4	0.452	7	0.318	3	0.131	-	-	2	0.801
	Somatic Variant	9	0.365	-	-	-	-	3	0.218	10	0.421	-	-
	**Integration**	**8**	**0.019**	**5**	**0.201**	5	0.225	2	0.246	10	0.319	2	0.113

**KIRC**	mRNA	2	0.176	2	0.176	7	0.073	2	0.219	9	0.072	2	0.176
	Methylation	3	0.111	3	0.111	6	0.128	3	0.577	10	0.14	3	0.111
	miRNA	2	0.138	2	0.138	5	0.509	2	0.138	-	-	2	0.138
	Somatic Variant	2	0.076	-	-	-	-	3	0.124	9	0.348	-	-
	**Integration**	**5**	**3×10**^**−3**^	**4**	**1.3×10**^**−4**^	6	0.104	3	0.248	7	0.067	2	0.176

## References

[1] Saria S, Goldenberg A. “Subtyping: What It is and Its Role in Precision Medicine”, IEEE Intelligent Systems (Volume: 30, Issue: 4, 7-8 2015)

[2] Rivenbark AG, O’Connor SM, Coleman WB, 2013 Molecular and Cellular Heterogeneity in Breast Cancer: Challenges for Personalized Medicine, The American Journal of Pathology, 183(4), 1113–1124. 23993780 10.1016/j.ajpath.2013.08.002 PMC5691324

[3] Vaz-Luis I, Winer EP, Lin NU, 2013 Human epidermal growth factor receptor-2-positive breast cancer: does estrogen receptor status define two distinct subtypes Annals of Onc, 24(2), 283–291. 10.1093/annonc/mds286 PMC3551479 23022997

[4] Hollander D, Savage PIM, & Brown PH 2013 Targeted Therapy for Breast Cancer Prevention, Frontiers in Oncology, 3, 250. 24069582 10.3389/fonc.2013.00250 PMC3780469

[5] Perou CM, Sørlie T, Eisen MB, van de Rijn M, Jeffrey SS, Rees CA, Pollack JR, Ross DT, Johnsen H, , 2000 Molecular portraits of human breast tumours. Nature 406: 747–752. 10963602 10.1038/35021093

[6] Eisen MB, Spellman PT, Brown PO, Botstein D, 1998 Cluster analysis and display of genome-wide expression patterns, Proc Natl Acad Sci 95: 14863–14868. 10.1073/pnas.95.25.14863 PMC24541 9843981

[7] Jiang D, Tang C, Zhang A, 2004 Cluster analysis for gene expression data: a survey. IEEE Trans Knowl Data Eng 16: 1370–1386.

[8] McLachlan GJ, Bean R, Peel D, 2002 A mixture model-based approach to the clustering of microarray expression data. Bioinformatics 18: 413–422. 11934740 10.1093/bioinformatics/18.3.413

[9] Sharan R, Shamir R. 2000 CLICK: a clustering algorithm with applications to gene expression analysis. In Proc Int Conf Intell Syst Mol Biol 8: 16 10977092

[10] Hartuv E. A clustering algorithm based on graph connectivity, Inform Pr. Lett 76:175–181.

[11] Chalise P, & Fridley BL, 2017 Integrative clustering of multi-level ‘omic data based on non-negative matrix factorization algorithm. doi:10.1371/journal.pone.0176278 PMC5411077 28459819

[12] Luo F, Khan L, Bastani F, Yen IL, Zhou J, 2004 A dynamically growing selforganizing tree for hierarchical clustering gene expression profiles. Bioinformatics 20: 2605–2617. 15130935 10.1093/bioinformatics/bth292

[13] Vasudevan P, Murugesan T. Cancer Subtype Discovery Using Prognosis-Enhanced Neural Network Classifier in Multigenomic Data. Technol Cancer Res Treat. 2018 1;17:1533033818790509. doi: 10.1177/1533033818790509. PMC6088521 30092720

[14] Greenman , 2007 Patterns of somatic mutation in human cancer genomes, Nature. 446(7132): 153–158. doi:10.1038/nature05610. 17344846 PMC2712719

[15] Vandin F, Upfal E, and Raphael BJ, 2011, Algorithms for detecting significantly mutated pathways in cancer, Journal of Computational Biology 18, 507. 21385051 10.1089/cmb.2010.0265

[16] Monti S, Tamayo P, Mesirov J, and Golub T. Consensus clustering: a resampling-based method for class discovery and visualization of gene expression microarray data. Machine Learning, 52(1–2):91–118, 2003.

[17] Shen R, Olshen AB, and Ladanyi M. Integrative clustering of multiple genomic data types using a joint latent variable model with application to breast and lung cancer subtype analysis. Bioinformatics, 25(22):2906–2912, 2009. 19759197 10.1093/bioinformatics/btp543 PMC2800366

[18] Mo Q, Wang S, Seshan VE, Olshen AB, Schultz N, Sander C, Powers RS, Ladanyi M, and Shen R. Pattern discovery and cancer gene identification in integrated cancer genomic data. Proceedings of the National Academy of Sciences, 110(11):4245–4250, 2013. 10.1073/pnas.1208949110 PMC3600490 23431203

[19] Wang B, Mezlini AM, Demir F, Fiume M, Tu Z, Brudno M, Haibe-Kains B, and Goldenberg A. Similarity network fusion for aggregating data types on a genomic scale. Nature Methods, 11(3):333–337, 2014. 24464287 10.1038/nmeth.2810

[20] Nguyen T, Tagett R, Diaz D, and Draghici S. A novel approach for data integration and disease subtyping. Genome Research, pages gr–215129, 2017. 10.1101/gr.215129.116 PMC5741060 29066617

[21] Gligorijević V, Malod-Dognin N, Pržulj N, “Integrative methods for analyzing big data in precision medicine”, Proteomics. 2016 3;16(5):741–58. doi: 10.1002/pmic.201500396. 26677817

[22] Muzny DM. , “Comprehensive molecular characterization of human colon and rectal cancer”, Cancer Genome Atlas Research Network, Nature 487, 330 (2012). doi: 10.1038/nature11252 22810696 PMC3401966

[23] Hofree M, Shen JP, Carter H, Gross A, Ideker T, “Network-based stratification of tumor mutations”, Nature Methods 10, 1108 (2013). 24037242 10.1038/nmeth.2651 PMC3866081

[24] Arslanturk S, Draghici S, ‘Disease Subtyping using Somatic Variant Data’, ACM Conference on Bioinformatics, Computational Biology, and Health Informatics 2018.

[25] Gouweleeuw JM “Post Randomisation for Statistical Disclosure Control: Theory and Implementation.” Journal of Official Statistics, Vol. 14, No. 4, 1998, pp. 463–478.

[26] Tibshirani R, Walther G, and Hastie T. Estimating the number of clusters in a data set via the gap statistic. Journal of the Royal Statistical Society: Series B (Statistical Meth.), 63(2):411–423, 2001.

[27] Parsons DW, Jones S, Zhang X, Lin JCH, Leary RJ, Angenendt P, 2008 An integrated genomic analysis of human glioblastoma multiforme. Science 321: 1807–1812. 18772396 10.1126/science.1164382 PMC2820389

[28] Wick W, Hartmann C, Engel C, Stoffels M, Felsberg J, Stockhammer F, Sabel MC, Koeppen S, Ketter R, Meyermann R, 2009 N0A-04 randomized phase III trial of sequential radiochemotherapy of anaplastic glioma with procarbazine, lomustine, and vincristine or temozolomide. J Clin Oncol 27: 5874–5880. 19901110 10.1200/JCO.2009.23.6497

[29] Huang D, Sun W, Zhou Y. Cancer Metastasis Rev (2018) 37: 173. 29322354 10.1007/s10555-017-9726-5

[30] Ramazzotti D, Lal A, Wang B, Batzoglou S & Sidow A. Multi-omic tumor data reveal diversity of molecular mechanisms that correlate with survival. Nature Communications 4453, (2018), DOI: 10.1038/s41467–018 −06921–8 10.1038/s41467-018-06921-8 PMC6203719 30367051

[31] Zhao Lan, Lee Victor H F, Ng Michael K, Yan Hong, Bijlsma Maarten F, Molecular subtyping of cancer: current status and moving toward clinical applications, Briefings in Bioinformatics, Volume 20, Issue 2, 3 2019, Pages 572–584 29659698 10.1093/bib/bby026

